# Impending Cardiac Tamponade as the Initial Manifestation of BCR-ABL Positive Chronic Myeloid Leukemia

**DOI:** 10.7759/cureus.9631

**Published:** 2020-08-09

**Authors:** Safa Moursy, Salem Gaballa, Ameenjamal Ahmed, Kyaw M Hlaing, Brijesh B Patel

**Affiliations:** 1 Internal Medicine, LewisGale Medical Center, Salem, USA; 2 Internal Medicine/Pulmonary and Critical Care, LewisGale Medical Center, Salem, USA

**Keywords:** cml, bcr-abl positive, cardiac tamponade, pericardial effusion, chronic myeloid leukemia

## Abstract

Leukemia involves all organs and tissues of the body. Leukemic infiltration of the pericardium has been documented frequently at post-mortem examinations. Clinically, however, pericardial effusion with cardiac tamponade is rare, and only isolated case reports have been described. In all the reported cases, therapeutic pericardiocentesis was required for the relief of cardiac tamponade with the risk of bleeding since these patients often had deranged hemostasis. We are reporting a rare case of hemorrhagic pericardial effusion in chronic myeloid leukemia before starting the tyrosine kinase inhibitors. The patient required therapeutic pericardiocentesis and hydroxyurea treatment.

## Introduction

Malignant involvement of the pericardium is seen in 1 to 20% of autopsies in patients with cancer. The most common metastatic tumor involving the pericardium is lung cancer; others include leukemia (4%), lymphoma (17%), breast (7.5%), and esophageal cancer (28%) [1]. Approximately 50% of chronic myeloid leukemia (CML) patients are asymptomatic at presentation. The most frequent complaints are fatigue, abdominal fullness, left upper quadrant fullness, and decreased exercise tolerance. Pericardial effusion in the chronic phase of CML is a rare occurrence, and association with tamponade is extremely rare. The proposed mechanism of the pericardial effusion may be related to leukemic infiltration, extramedullary hematopoiesis, infections, and bleeding in CML.

## Case presentation

A 69-year-old female with a recent diagnosis of BCR/ABL+ CML, type 2 diabetes mellitus, hypertension, and coronary artery disease presented to the ED with complaints of shortness of breath (SOB). The patient reported her SOB started worsening over the last couple of days prior to admission. She denied any orthopnea or paroxysmal nocturnal dyspnea. She also reported dizziness (positional), palpitations, poor oral intake due to poor appetite, and nausea. The patient reported several episodes of coffee-ground emesis and black tarry stools for the last couple of days. She denied any hematemesis or bright red blood per rectum (BRBPR). She also endorsed easy bruising and episodes of blurry vision. Initial vital signs in the ED showed a heart rate of 105 beats per minute, blood pressure of 89/55, and 85% saturation on 4L nasal cannula. Lung exam revealed crackles with decreased breath sounds, muffled heart sounds, and no murmurs.

**Figure 1:**
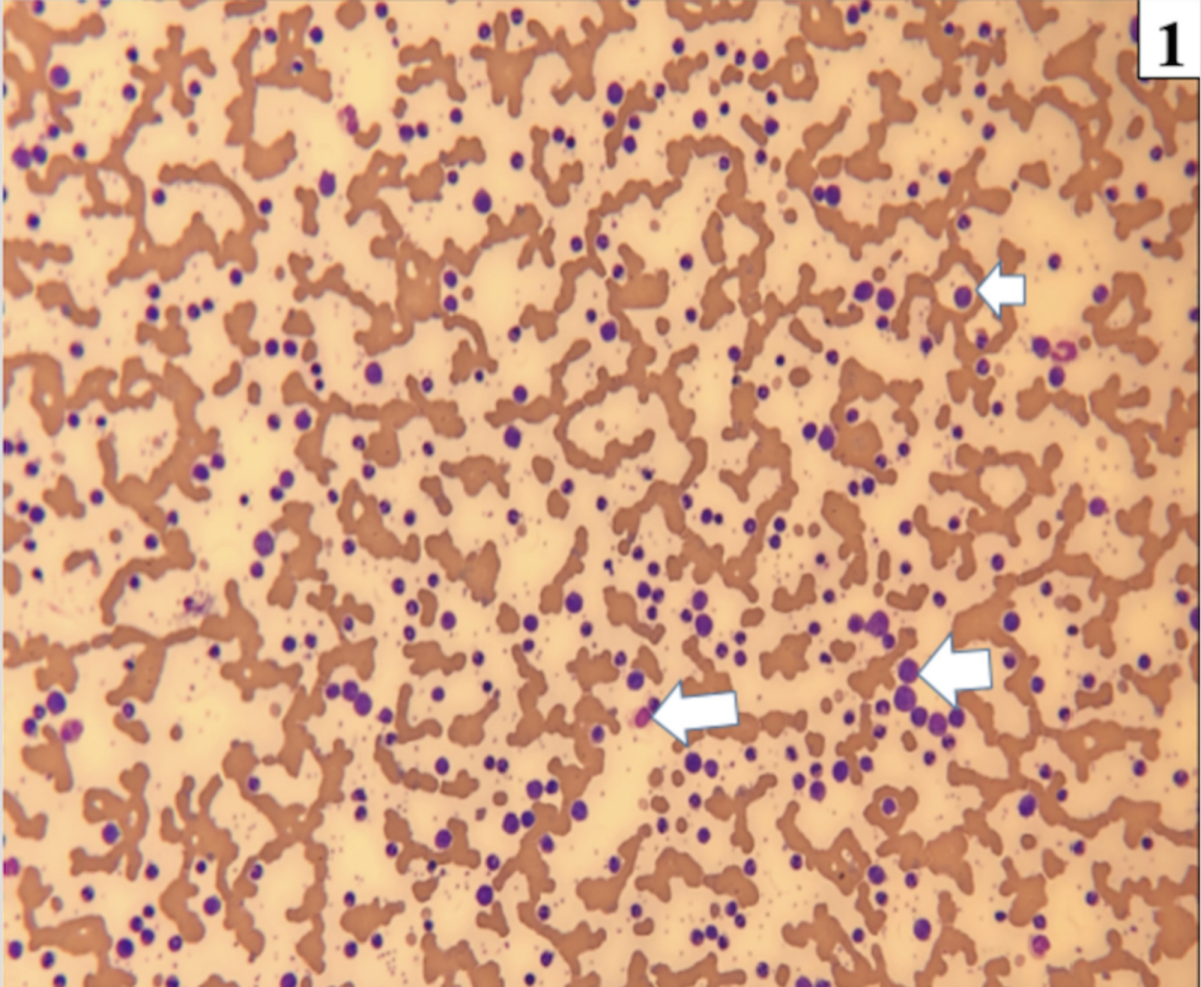
Blood film at 100x magnification demonstrates leukocytosis with the presence of precursor cells of the myeloid lineage (white arrows). In addition, basophilia, eosinophilia, and thrombocytosis is are seen.

Lab results were as follows: hemoglobin of 7.9 g/dL, hematocrit of 25.6%, platelets of 379 K/mm^3^, WBCs of 105.2 K/mm^3^, and peripheral smear (Figure 1) showed neutrophils of 46%, bands of 10%, lymphocytes of 10%, monocytes of 4%, metamyelocytes of 21%, myelocytes of 9%, and blasts of 1%.

Complete metabolic profile showed Na (sodium) of 134 mmol/L, K (potassium) of 7.3 mmol/L, bicarbonate of 19 mmol/L, BUN (blood urea nitrogen) of 103 mg/dL, creatinine of 3.4 mg/dL, glucose of 208 mg/dL, AST (aspartate aminotransferase) of 17 U/L, ALT (alanine aminotransferase) of 12 U/L, uric acid of 15.2 mg/dL, proBNP (pro b-type natriuretic peptide) of 1,135 pg/mL, troponin of <0.015 ng/mL, and procalcitonin of 1.3 ng/mL. COVID-19 screen was negative. Blood culture showed no growth after five days. Immunoglobulins were within normal limits. RF and ANA screen was negative. P-ANCA and C-ANCA were negative. C3 and C4 were within normal limits.

**Figure 2:**
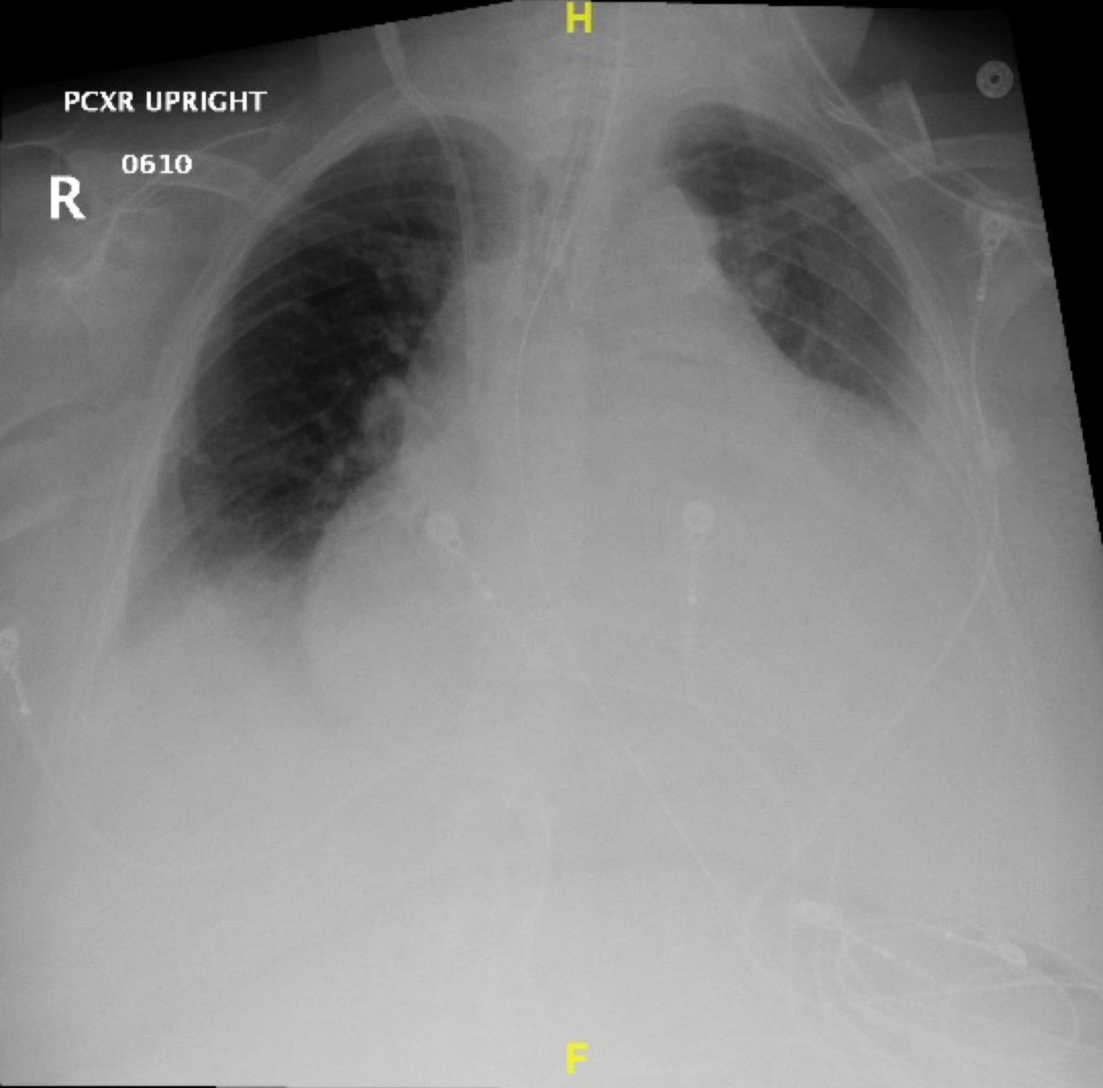
CXR showing left-sided pleural effusion with a water bottle sign concerning for pericardial effusion. CXR, chest X-ray

**Figure 3:**
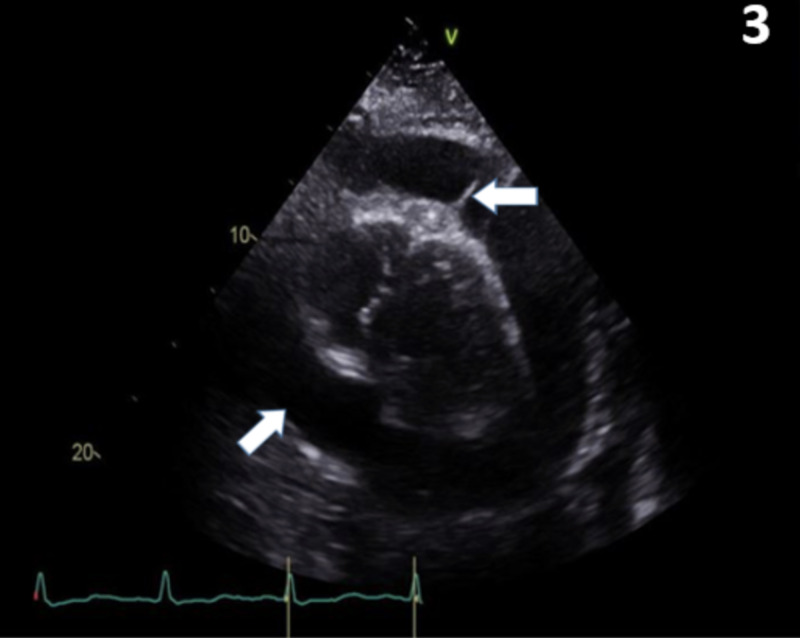
Transthoracic echocardiography shows a large (2 cm) pericardial effusion circumferentially surrounding the heart with fibrinous focal strands (white arrows).

Chest X-ray (Figure 2) showed air space disease, left side pleural effusion, and a boot-shaped heart concerning for pericardial effusion. Transthoracic echocardiography showed a large pericardial effusion of 2 cm circumferentially surrounding the heart with focal strands. There were no echo signs of frank tamponade (Figure 3).

**Figure 4:**
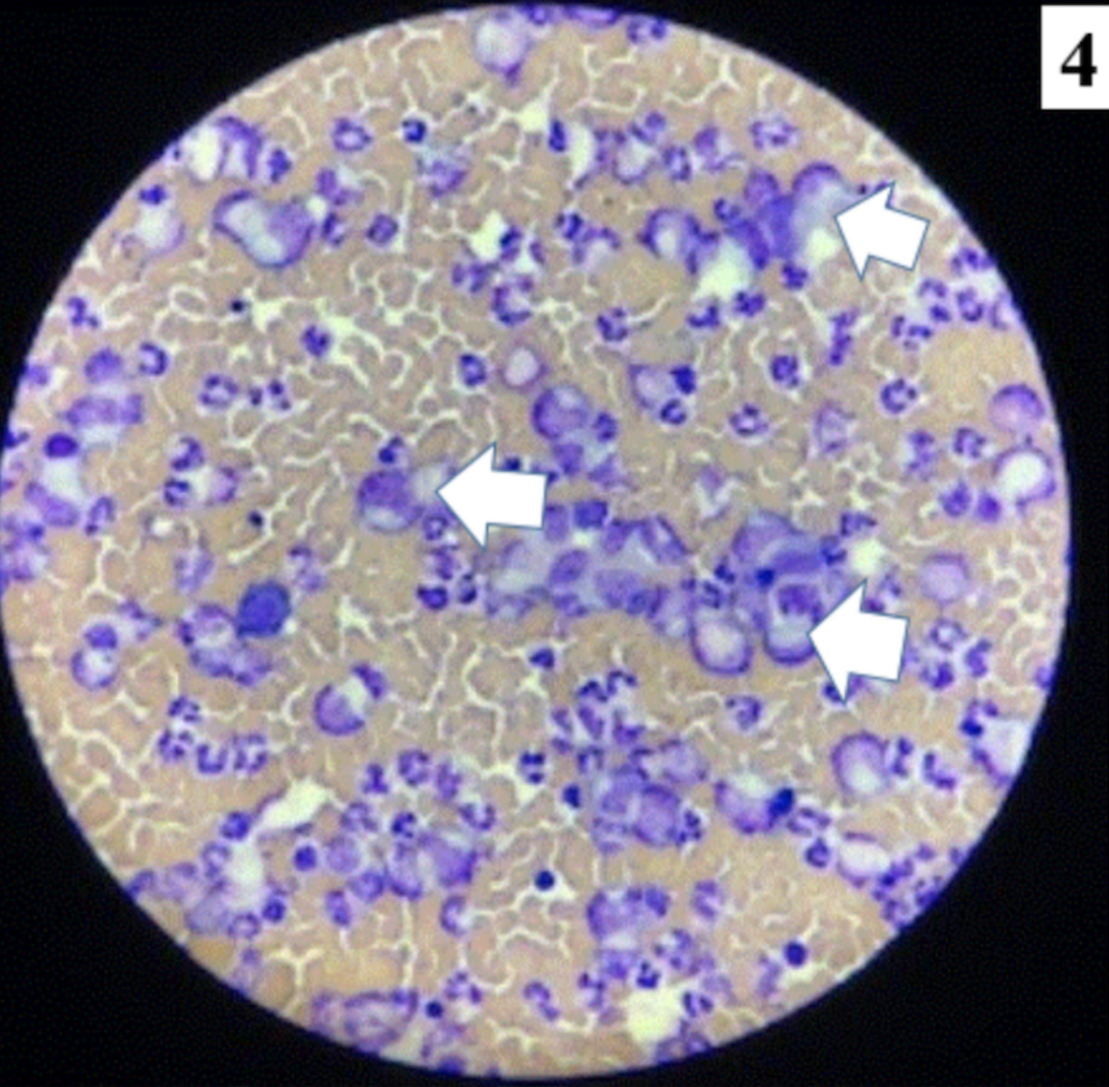
Microphotograph of the pericardial cytology showing numerous myelocytes, neutrophils, and smudge cells against a hemorrhagic background (white arrows).

Bedside pericardiocentesis was performed and 900 cc of frank bloody fluid was removed. A drain was left in place for subsequent removal of 400 cc on day 2 and 250 cc on day 3. Pericardial fluid analysis revealed the following: negative gram stain/culture, bloody appearance, 2,300,000 RBCs, and 64,420 WBCs with polymorphonuclear neutrophils (PMNs) of 94%, lymphocytes of 2%, and monocytes of 2%. Pericardial cytology shows infiltration by CML (Figure 4).

The patient was treated in the critical care unit for multifocal pneumonia, severe sepsis, and acute renal failure requiring hemodialysis (HD). The patient's hemodynamics improved after pericardiocentesis, and a total of 1,550 cc of bloody pericardial fluid was removed. WBCs decreased to 85k with hydroxyurea. Tyrosine kinase inhibitors (TKIs), such as nilotinib, were not started due to prolonged QTc (550 ms) and resistant atrial fibrillation. The patient was instructed to follow up with the outpatient hematology/oncology service to start chemotherapy once stable.

## Discussion

Malignancy is a common cause of large symptomatic pericardial effusions [2,3], with symptomatic effusions being defined as those with cardiorespiratory symptoms (e.g., dyspnea), signs (e.g., tachycardia), echocardiographic features of right heart compromise, or if pericardiocentesis was deemed therapeutic by the clinician. In some cases, the effusion may be the initial clinical manifestation of malignancy. Importantly, pericardial effusions in patients with cancer may also be triggered by a mechanism other than cancer itself, including chemotherapy and radiation therapy. Traditional chemotherapy agents such as fludarabine, cytarabine, doxorubicin, docetaxel, and cyclophosphamide have been associated with acute pericarditis and pericardial effusion. Dasatinib, a TKI targeting BCR-ABL, KIT, and platelet-derived growth factor receptor-beta, has also been linked to an increased incidence of pericardial effusions [4].

Most patients without a hemodynamically significant pericardial effusion (i.e., without cardiac tamponade) will have no symptoms specific to the effusion, but they may have symptoms related to the underlying cause (e.g., fever in the setting of pericarditis). The clinical features of cardiac tamponade usually depend upon whether the onset of fluid accumulation is acute or subacute; cardiac tamponade is usually subacute in the setting of malignancy.

Pericardiocentesis with cytological and/or flow cytometry examination of the pericardial fluid should be performed in patients with hemorrhagic pericardial effusion whenever there is a reason to suspect malignancy. Cytological evaluation is especially critical if the effusion is hemorrhagic, and there is no history of antecedent trauma; such effusions are more likely to be malignant rather than non-hemorrhagic [5]. The sensitivity of cytology for the diagnosis of a malignant effusion is between 67% and 92%. Positive cytology may be associated with poor outcomes in patients with neoplastic pericardial disease. Patients with positive cytology were also more likely to require repeat pericardiocentesis or surgical intervention [6]. There are several proposed mechanisms of pericardial effusions in patients with CML. These include the following:

A. Leukemic infiltration into the pericardium that usually occurs at the time of or just prior to bone marrow evolution to blast crisis phase [7]. In these cases, the pericardial fluid contains a higher proportion of blast cells. Leucocyte alkaline phosphatase (LAP) activity, known to be low in CML granulocytes of peripheral blood, has been reported to be normal in the granulocytes of the pericardial effusion. Additionally, in Philadelphia chromosome-positive cases, the Philadelphia chromosome is detected in the pericardial granulocytic cells by conventional cytogenetic methods [8].

B. Extramedullary hematopoiesis, although the pericardium, is are rarely a site in CML patients. [7] Unlike pericardial leukemic infiltration, extramedullary hematopoiesis includes hematopoietic cells of the erythroid, myeloid, and megakaryocytic cells, although one linkage can predominate [9].

C. Obstruction of pericardial capillaries or infiltration of interstitial tissue by leukemic cells during uncontrolled leukocytosis and increased capillary permeability due to cytokine production [7]. Predisposing factors such as leukostasis and platelet dysfunction may have a role in the hemorrhagic effusion of CML. Leukostasis can cause plugging of blood vessels with secondary hemorrhage. Marked thrombocytosis and abnormal platelet function in CML may also add to it [9].

D. Non-malignant etiologies, including infection and hypoproteinemia, have also been postulated as the cause of the effusion. Therefore, this possibility must be excluded by the identification of microorganisms by special stain and/or the presence of necrotic debris [7]. E. Drug-induced through TKIs, dasatinib and imatinib, have significant anti-leukemic activity in CML patients. Their use has been associated with pericardial effusion in 15% of cases in one study [10].

Management of highly symptomatic patients or those with evidence of hemodynamic compromise requires urgent fluid removal to alleviate symptoms and prevent hemodynamic collapse. The fluid is typically removed either by percutaneous pericardiocentesis under echocardiographic guidance or at the time of surgical creation of a pericardial window. This generally results in a rapid and dramatic improvement in symptoms and hemodynamics, even if clinical or echocardiographic signs of cardiac tamponade persist. Prolonged catheter drainage effectively prevents fluid reaccumulation, although the mechanism by which this occurs is unclear. If rates of fluid drainage are still high after three to five days, a pericardial window should be considered [11,12].

Most patients with an asymptomatic malignant pericardial effusion have a short life expectancy [13]. However, the prognosis may be better in specific subsets of patients, such as those without cancerous cells in the pericardium, hematologic rather than solid tumors, patients who are candidates for systemic therapy, or those whose malignancy is otherwise well controlled [14].

## Conclusions

Pericardial effusion should be considered in patients with leukemia who experience a sudden onset of cardiac symptoms. Management should, however, be individualized. In those in whom the pericardial effusion is the initial presentation of the disease and in case the clinical status of the patients allows, pericardiocentesis may be withheld and systemic chemotherapy be promptly administered, thus avoiding the potential complications of pericardiocentesis in patients with a bleeding tendency. Pericardiocentesis is required when the pericardial effusion is resistant to chemotherapy, or when patient symptoms are severe, and when an infection has to be excluded. We also emphasize the fact that there is significant research going on at present on the new drugs for CML, imatinib and dasatinib, which have shown that pericardial effusion is being seen very frequently after starting these drugs. This case brings to notice the need for a two-dimensional echo prior to starting with the newer medications for CML to rule out pre-existent effusion.
